# A two-step immunoassay for the simultaneous assessment of Aβ38, Aβ40 and Aβ42 in human blood plasma supports the Aβ42/Aβ40 ratio as a promising biomarker candidate of Alzheimer’s disease

**DOI:** 10.1186/s13195-018-0448-x

**Published:** 2018-12-08

**Authors:** Hedieh Shahpasand-Kroner, Hans-W. Klafki, Chris Bauer, Johannes Schuchhardt, Melanie Hüttenrauch, Martina Stazi, Caroline Bouter, Oliver Wirths, Jonathan Vogelgsang, Jens Wiltfang

**Affiliations:** 1 Department of Psychiatry and Psychotherapy, University Medical Center Goettingen (UMG), Georg-August-University, Von-Siebold-Str. 5, D-37075 Goettingen, Germany; 2 German Center for Neurodegenerative Diseases (DZNE), Von-Siebold-Str. 3a, D-37075 Goettingen, Germany; 3 Present address: Department of Neurology, David Geffen School of Medicine, Neuroscience Research Building, University of California at Los Angeles, Los Angeles, CA 90095 USA; 4 MicroDiscovery GmbH, Marienburger Strasse 1, D-10405 Berlin, Germany; 5 Department of Nuclear Medicine, University Medical Center Goettingen (UMG), Georg-August-University, Robert-Koch-Str. 40, D-37075 Goettingen, Germany; 6 Department of Medical Science, iBiMED, University of Aveiro, Aveiro, Portugal

**Keywords:** Alzheimer’s disease, Dementia, Amyloid beta, Blood plasma, Cerebrospinal fluid, Biomarker, Immunoprecipitation

## Abstract

**Background:**

The quantification of amyloid-beta (Aβ) peptides in blood plasma as potential biomarkers of Alzheimer’s disease (AD) is hampered by very low Aβ concentrations and the presence of matrix components that may interfere with the measurements.

**Methods:**

We developed a two-step immunoassay for the simultaneous measurement of the relative levels of Aβ38, Aβ40 and Aβ42 in human EDTA plasma. The assay was employed for the study of 23 patients with dementia of the Alzheimer’s type (AD-D) and 17 patients with dementia due to other reasons (OD). We examined relationships with the clinical diagnosis, cerebral Aβ load as quantified by amyloid-positron emission tomography, apolipoprotein E genotype, Aβ levels and Tau protein in cerebrospinal fluid.

**Results:**

Preconcentration of plasma Aβ peptides by immunoprecipitation substantially facilitated their immunological measurements. The Aβ42/Aβ40 and Aβ42/Aβ38 ratios were statistically significantly lower in the AD-D patients than in the OD group. The areas under the receiver operating characteristic curves reached 0.87 for the Aβ42/Aβ40 ratio and 0.80 for the Aβ42/Aβ38 ratio.

**Conclusions:**

The measurement of plasma Aβ peptides with an immunological assay can be improved by preconcentration via immunoprecipitation with an antibody against the Aβ amino-terminus and elution of the captured peptides by heating in a mild detergent-containing buffer. Our findings support the Aβ42/Aβ40 ratio in blood plasma as a promising AD biomarker candidate which correlates significantly with the validated core biomarkers of AD. Further studies will be needed for technical advancement of the assay and validation of the biomarker findings.

**Electronic supplementary material:**

The online version of this article (10.1186/s13195-018-0448-x) contains supplementary material, which is available to authorized users.

## Background

Neurochemical biomarkers in cerebrospinal fluid (CSF) and imaging biomarkers can support the early diagnosis of Alzheimer’s disease (AD). In numerous studies, low CSF concentrations of the amyloid-beta peptide Aβ42 and elevated CSF levels of total Tau (tTau) and phosphorylated Tau (pTau) have been reported in patients with Alzheimer’s disease dementia or mild cognitive impairment due to AD [1, 2]. The reduction of CSF Aβ42 in AD is negatively correlated with ^11^C-Pittsburgh compound B uptake on in-vivo amyloid positron emission tomography (PET) imaging [3, 4], and appears to reflect cerebral amyloid accumulation. Other possible theoretical explanations for the selective decrease in measurable, soluble CSF Aβ42 under pathological conditions include decreased Aβ production, increased Aβ42 degradation, increased clearance from the brain into the blood, and Aβ oligomerization leading to epitope masking (reviewed in [2]). Increased CSF tau levels in AD (both tTau and pTau) are considered biomarkers of neuronal destruction [5]. The development of a robust and reliable blood-based biomarker assay of AD neuropathology applicable to high-throughput clinical routine and clinical trial recruitment would be highly advantageous. Blood can be sampled by minimally invasive procedures that are well accepted by the patients, and is suitable for longitudinal studies requiring multiple measures. The diagnostic potential of Aβ peptides in blood plasma has been the subject of numerous studies. These have produced conflicting results, and thus the authors of a recent comprehensive meta-analysis and review concluded that there were no significant differences in Aβ42 or Aβ40 levels in blood plasma or serum between Alzheimer’s disease patients and controls, in general [1]. However, two very recent studies employing pre-enrichment of plasma Aβ peptides by immunoprecipitation (IP) followed by mass spectrometry (MS) showed that the concentration ratios of Aβ42/Aβ40, Aβ1–40/Aβ1–42 and APP669–711/Aβ1–42 can predict the presence of cerebral Aβ amyloidosis with very high accuracy [6, 7]. It has been estimated that approximately 30–50% of the Aβ peptides in blood plasma originate from the brain [6]. Thus, it is conceivable that the selective decrease in soluble CSF Aβ42 in AD may be detectable in plasma with highly sensitive, accurate and robust assays. The Aβ levels in blood plasma are roughly 50–100 times lower than those in CSF [8, 9], making their reliable measurement technically very challenging. Furthermore, matrix components present in blood plasma and serum may cause interferences (“matrix effects”) contributing to errors or incorrect results in immunoassays [10]. These technical challenges may explain, at least in part, the contradictory findings of previous studies addressing the diagnostic potential of Aβ peptides in blood as AD biomarker candidates.

In the present study, we have investigated whether immunoprecipitation of plasma Aβ peptides may facilitate the quantitative assessment of Aβ peptide ratios by a sandwich-type immunological multiplex assay. We report on the development of a prototype two-step immunoassay protocol for the simultaneous measurement of the relative concentrations of Aβ38, Aβ40 and Aβ42 in human blood plasma. Aβ peptides were first preconcentrated by immunoprecipitation with functionalized magnetic beads carrying an antibody against the Aβ N-terminus and then analyzed with a multiplex electrochemiluminescence sandwich immunoassay. Importantly, the elution of the captured Aβ peptides from the magnetic bead immune complexes was achieved by heating in a buffer that is compatible with the subsequent Aβ sandwich immunoassay. In a clinical sample in which the diagnosis was cross-validated by cranial computed tomography (cCT) or magnetic resonance imaging (MRI) (*n* = 40), CSF dementia biomarkers (CSF Aβ1–42, *n* = 37; CSF Aβ1–42/Aβ1–40 ratio, *n* = 36; CSF tTau, *n* = 37; CSF pTau181, *n* = 37), amyloid-beta positron emission tomography (amyloid-PET) (*n* = 18) and ^18^F-fluorodeoxyglucose positron emission tomography (^18^F-FDG-PET) (*n* = 20), we observed statistically significant differences in the plasma Aβ42/Aβ40 and Aβ42/Aβ38 ratios between patients with probable or possible AD dementia and dementia due to other reasons.

## Materials and methods

### Study approval and study cohort

The participants of the study were recruited in the Department of Psychiatry and Psychotherapy at the University Medical Center Goettingen, Georg-August-University between July 2016 and September 2017. The collection and archiving of biological samples and clinical data in strictly pseudonymous form in a local biobank and their use in biomarker research were approved by the ethics committee of the University Goettingen (9/2/16). Written informed consent was obtained prior to inclusion in the biobank from all participants or their legal representatives. The study cohort was essentially identical to that described previously [11] and comprised 40 subjects who fulfilled the clinical criteria of dementia. Dementia was diagnosed according to the 2011 McKhann criteria, and diagnosis did consider psychometric testing and the patient’s ability to function in usual daily activities [12]. For the majority of the study participants, the CSF-biomarkers total Tau (tTau), phospho Tau181 (pTau), Aβ1–42 and the Aβ1–42/Aβ1–40 ratio were determined in the Neurochemical Laboratory at the University Medical Center Goettingen, Germany. The CSF measurements of tTau, pTau and Aβ1–42 were done with ELISA kits obtained from Fujirebio, and Aβ1–40 was determined with an ELISA kit obtained from IBL International. The diagnostic cutoff points were 450 pg/ml for tTau, 61 pg/ml for pTau, 450 pg/ml for Aβ1–42 and 0.05 for the Aβ1–42/Aβ1–40 ratio.

All patients received cerebral imaging (cCT or cMRI). ^18^F-FDG-PET data and amyloid-PET data were available for 20 and 18 of the study participants, respectively. Quantifiable amyloid-PET data were available for 16 of the study participants. All PET/CT studies were performed on a Philips Gemini TF16 PET/CT-scanner (Philips Medical Systems, Cleveland, OH, USA) with a 128 × 128 matrix with 2-mm slice thickness. Low-dose CT was used for attenuation correction and images were reconstructed with the LOR-TF-RAMLA (“BLOB-OS-TF”) algorithm. For ^18^F-FDG-PET/CT, 150 MBq (± 10%) ^18^F-FDG was administered intravenously. Patients should have fasted for at least 4–6 h in advance and blood glucose levels had to be below 150 mg/dl. All patients rested in a quiet, dimly-lit room for at least 30 min before and for another 20 min after injection of the tracer. Image acquisition was performed 60 min post injection. The acquisition time was 10 min. PET/CT scans were visually assed by an experienced nuclear medicine physician. Images were evaluated with reference to anatomic brain images of CT and semi-quantitative analysis was performed obtaining standardized uptake values (SUV) of regional cortical areas.

Amyloid imaging was performed by administering 300 (± 20%) MBq ^18^F-florbetaben (Piramal Imaging Ltd, Cambridge, UK) intravenously. PET/CT was performed 90 min post injection with an acquisition time of 20 min. PET/CT scans were visually assed by an experienced nuclear medicine physician who underwent a specific reader training for amyloid imaging. Amyloid uptake was assed semi-quantitatively using CortexID Suite (GE Healthcare, Little Chalfont, UK) with automated gray matter segmentation using a T1-weighted MRI template. Standardized uptake values of different cortical areas were obtained and standardized uptake value ratios (SUVr) were calculated using the whole cerebellum as the reference.

Fifteen of the patients included in this study were diagnosed as having probable AD dementia, eight as having possible AD dementia with AD pathology (mixed vascular and Alzheimer’s dementia) and 17 as having dementia due to other reasons (other dementias (OD)). For a dichotomized biostatistical data evaluation, the patients with probable AD dementia and those with possible AD dementia were combined into the single diagnostic category dementia of the Alzheimer type (AD-D). Whenever available, CSF biomarkers Aβ1–42 (*n* = 37), Aβ1–42/Aβ1–40 ratio (*n* = 36), tTau (n = 37) and pTau181 (n = 37) or positron emission tomography (PET) scans (amyloid-PET, *n* = 16 and ^18^FDG-PET, *n* = 20) were considered to support the clinical diagnosis.

The patients categorized as AD-D showed biomarker evidence for cerebral amyloid pathology (low CSF Aβ1–42, low CSF Aβ1–42/Aβ1–40 ratio or increased tracer uptake on amyloid PET) and abnormal CSF-Tau or FDG-PET signals. Evidence of brain amyloid was absent in the OD group.

The characteristics of the study cohort are summarized in Table 1.

**Table 1:** Characteristics of the study cohort

Characteristic	Dementia of the Alzheimer type	Dementia due to other reasons	*p* value
Number	23	17	
Gender (male/female)	10/13	12/5	0.116*
Age (years)	69.0 ± 10.9	64.4 ± 11.0	0.199**
MMSE	22.6 ± 4.4	25.3 ± 2.4	0.041**
CDT (points)	3.0 ± 1.3	2.4 ± 1.2	0.147**
CSF Aβ1–40 (ng/ml)	11.87 ± 3.67 (*n* = 21)	10.08 ± 3.43 (*n* = 15)	0.147**
CSF Aβ1–42 (ng/ml)	0.59 ± 0.15 (*n* = 22)	1.20 ± 0.28 (*n* = 15)	< 0.0001**
CSF Aβ42/40 ratio	0.052 ± 0.18 (*n* = 21)	0.126 ± 0.30 (*n* = 15)	< 0.0001**
CSF tTau (pg/ml)	731.6 ± 283.6 (*n* = 22)	300.8 ± 116.5 (*n* = 15)	< 0.0001**
CSF pTau (pg/ml)	85.1 ± 32.0 (*n* = 22)	42.5 ± 12.2 (*n* = 15)	< 0.0001**

Data presented as mean ± standard deviation, unless otherwise stated

*Aβ* amyloid beta, *CDT* clock-drawing test, *CSF* cerebrospinal fluid, *MMSE* Mini-Mental State Examination, *pTau* phosphorylated Tau protein, *tTau* total Tau protein

*Fisher’s exact test

**Unpaired *t* test for differences between the two diagnostic categories

### Sample preparation and storage

Blood samples were collected in EDTA Monovettes (S-Monovette 9 ml K3E; Sarstedt, Nümbrecht, Germany) and centrifuged for 10 min at 2000 × *g* at room temperature to obtain EDTA blood plasma. Until use, EDTA plasma was stored in 500 μl aliquots in polypropylene tubes at − 80 °C.

### Apoliprotein E genotyping

Apolipoprotein E (*APOE*) genotyping was carried out using a real-time polymerase chain reaction (PCR) protocol as described previously by Calero et al. [13]. In brief, the four different primers ApoE_112C (5′-CGGACATGGAGGACGTGT-3′), ApoE_112R (5′-CGGACATGGAGGACGTGC-3′), ApoE_158C (5′-CTGGTACACTGCCAGGCA-3′) and ApoE_158R (5′-CTGGTACACTGCCAGGCG-3′) were obtained from Eurofins Genomics and combined in three different reaction mixtures: *APOE* ε2 (primers ApoE_112C and ApoE158C), *APOE* ε3 (primers ApoE_112C and ApoE_158R) and *APOE* ε4 (primers ApoE_112R and ApoE_158R). All PCR reactions were run in duplicate including negative controls for each primer combination. Following an initial polymerase activation at 95 °C for 10 min, 40 cycles consisting of a denaturation step at 95 °C for 15 s and a combined annealing and extension phase at 62 °C for 1 min were run followed by a subsequent melting curve analysis on a Stratagene MX3000P Real-Time PCR instrument.

### Preparation of functionalized anti-Aβ magnetic beads

To prepare 1 ml of functionalized magnetic particles, Dynabeads™ M-280 Sheep Anti-Mouse IgG (6 × 10^8^–7 × 10^8^ beads/ml; ThermoFisher Scientific) were resuspended in the original glass container, and 1 ml was transferred into a polypropylene reaction vial. The vial was placed on a magnetic stand for a few minutes, and the supernatant was carefully removed and discarded. The beads were washed twice with 1 ml of PBS/0.1% (w/v) BSA for 3 min at room temperature on a mixer. The washed beads were resuspended in 1 ml of PBS/0.1% BSA and 40 μg of monoclonal anti-Aβ antibody clone 1E8 (nanoTools GmbH, Teningen, Germany) was added. The mixture was incubated for 2 h at room temperature or overnight at 4 °C on a mixer. Then, the beads were washed 4× for 30 min at 4 °C and 1× for 5 min at room temperature with 1 ml of PBS/0.1% BSA. For covalent crosslinking, the beads were washed 2× for 5 min with 1 ml of 0.2 M triethanolamine and incubated for 1 h at room temperature on a mixer with 1 ml of 0.5% (w/v) dimethylpimelidate in 0.2 M triethanolamine. To stop the reaction, the tube was placed on a magnetic stand, the supernatant was removed and 1 ml of 50 mM Tris/HCl, pH 7.5, was added. After 5 min of incubation at room temperature on a mixer, the supernatant was discarded and the beads were washed three times with 1 ml of PBS/0.1% BSA for 5 min. The functionalized beads were stored until use at 4 °C in 1 ml of PBS/0.1% BSA/0.02% NaN_3_.

### Preconcentration of Aβ peptides from plasma by immunoprecipitation

Starting from an immunoprecipitation protocol we have described previously [9, 14], we tested modifications of the original procedure, such as different volumes of EDTA plasma as starting material, different volumes of elution buffer (see later) and different sample dilutions in the downstream Aβ measurements. In the final protocol applied to the assessment of the clinical cohort, 400 μl of each EDTA blood plasma sample was mixed with 100 μl of 5× immunoprecipitation (IP) buffer concentrate (250 mM HEPES/NaOH, pH 7.4, 750 mM NaCl, 2.5% Igepal CA630, 1.25% sodium deoxycholate; 0.25% SDS and 1 tablet of Complete Mini Protease inhibitor cocktail (Roche Diagnostics GmbH, Mannheim, Germany) per 2 ml). Then, 25 μl of functionalized anti-Aβ magnetic beads was added to each sample, and the mixtures were incubated overnight at 4 °C on a mixer. On the next day, the supernatants were discarded and the magnetic-bead immune complexes were washed 3× for 5 min with 1 ml of PBS/0.1% BSA and 1× for 3 min with 1 ml of 10 mM Tris/HCl, pH 7.5. Immediately before the Aβ measurements by the multiplex immunoassay (see later), the Aβ peptides were eluted in 30 μl of 20 mM bicine, 0.6% CHAPS (3-((3-cholamidopropyl)dimethylammonio)-1-propanesulfonate), pH 7.6, by heating to 95 °C for 5 min [14]. For the simultaneous measurement of Aβ38, Aβ40 and Aβ42 in quadruplicate reads, 25 μl of each IP eluate was mixed in a separate vial with 95 μl of Diluent 35 (Mesoscale Discovery).

### Chemiluminescence immunomultiplex assay

The Aβ38, Aβ40 and Aβ42 levels in the IP eluates were measured with the V-Plex Aβ panel 1 (6E10) multiplex assay kit (Mesoscale Discovery (MSD)) immediately after elution from the magnetic beads and 4.8-fold dilution with Diluent 35 (see earlier). Following the manufacturer’s instructions, the assay plate was blocked with 150 μl Diluent 35 per well for 1 h at room temperature on a mixer at 300 rpm and subsequently washed 3× with 150 μl PBS containing 0.05% Tween-20 (PBS-T) per well. The detection antibody solution was prepared for one plate by mixing 60 μl of the Sulfo-Tag 50× 6E10 antibody, 30 μl of Aβ40 blocker and 2910 μl Diluent 100 (all three reagents provided in the assay kit). Then, 25 μl of detection antibody solution and 25 μl of diluted sample or calibrator were pipetted into the wells of the assay plate. The plate was sealed and incubated for 2 h at room temperature on a shaker. After 3× washing with 150 μl of PBS-T per well, 150 μl of 2× read buffer was added to each well and the plate was read on a MESO QuickPlex SQ 120 reader (Mesocale Discovery). The lower limits of quantification (LLOQs) according to the certificates of analysis provided with the assay kits were 50 pg/ml (Aβ40), 60 pg/ml (Aβ38) and 3.13 pg/ml (Aβ42). The lower limit of detection (LLOD) was calculated with the MSD Discovery Workbench software automatically for each assay plate as the lowest concentration producing a signal three standard deviations above the zero calibrator with the option “use minimum error estimates” activated [15].

### Statistics and data evaluation

For statistical evaluation we used Graph Pad Prism 6.07 and R version 3.4.0. For the calculation of single-value receiver operating characteristic (ROC) curves, areas under the curves (AUCs) and the comparison of ROC curves we used the pROC package (https://www.ncbi.nlm.nih.gov/pubmed/21414208) version 1.9.1 and Graph Pad Prism 6.07. To calculate *p* values for the AUCs of the ROC curves, Prism assumes “that the area is really 0.5 (null hypothesis)”. In that case, the standard error (SE) is computed according to the simplified equation:

$$ \left(\mathrm{SE}\right)\kern0.5em =\kern0.5em \sqrt{\frac{0.25+\left(\kern0em na+ nn\hbox{-} 2\right)}{na^{\ast}\;{nn}^{\ast}\;12}} $$

where *na* = number of abnormals and *nn* = number of normals. Then, a *z* ratio is computed using the equation:

$$ z=\frac{A-0.5}{SE} $$

where A = area, and the *p* value is determined from the normal distribution (two-tail) (https://www.graphpad.com/guides/prism/6/statistics, calculation details for ROC curves; accessed 8 October 2018 [16].

Correlation coefficients were calculated according to Pearson and significance was calculated with a *t* statistic. For linear regression we used Deming regression since we assume that both variables are measured with error (R-package MethComp version 1.22.2). Comparison of the three *APOE* ε4 groups (zero, one or two *APOE* ε4 alleles) was done using an *F* test. Normality of a distribution was tested with the Shapiro–Wilk test.

## Results

### Immunoprecipitation facilitates the subsequent immunological measurement of the relative plasma levels of Aβ38, Aβ40 and Aβ42

In a series of pilot experiments, we tested the hypothesis that pre-enrichment by magnetic bead immunoprecipitation could substantially facilitate the semi-quantitative measurement of plasma Aβ38, Aβ40 and Aβ42 by the V-Plex Aβ panel 1 (6E10) multiplex assay kit (Mesoscale Discovery). To this end, five different EDTA-plasma samples were analyzed*,* either after 4-fold dilution in Diluent 35 or after Aβ preconcentration by magnetic bead immunoprecipitation. We tested immunoprecipitation (IP) from 400 or 800 μl of EDTA plasma followed by elution of preconcentrated Aβ from the immune complexes at 95 °C in bicine-CHAPS buffer. The IP eluates were diluted 10-fold or 20-fold with Diluent 35 and measured in duplicate with the triplex immunoassay. After 4-fold dilution of the plasma (without IP), five out of five tested samples showed Aβ42 signals in the quantitative range of the assay (i.e., above the LLOQ of the kit lot, as indicated by the manufacturer).

The Aβ40 signals in all five diluted plasma samples were above the LLOD of the tested assay plate. However, only two of the five tested diluted plasma samples showed Aβ40 signals above the LLOQ in both technical replicates, and were thus clearly in the quantitative range of the assay. In four out of five diluted plasma samples, the Aβ38 signals were below the detection range of the assay plate. The fifth sample showed a signal above the LLOD but below the LLOQ. Taken together, in our hands, the sensitivity of the triplex assay kit was not sufficient to reliably quantify all three Aβ peptides directly in 4-fold diluted blood plasma.

**Fig. 1:**
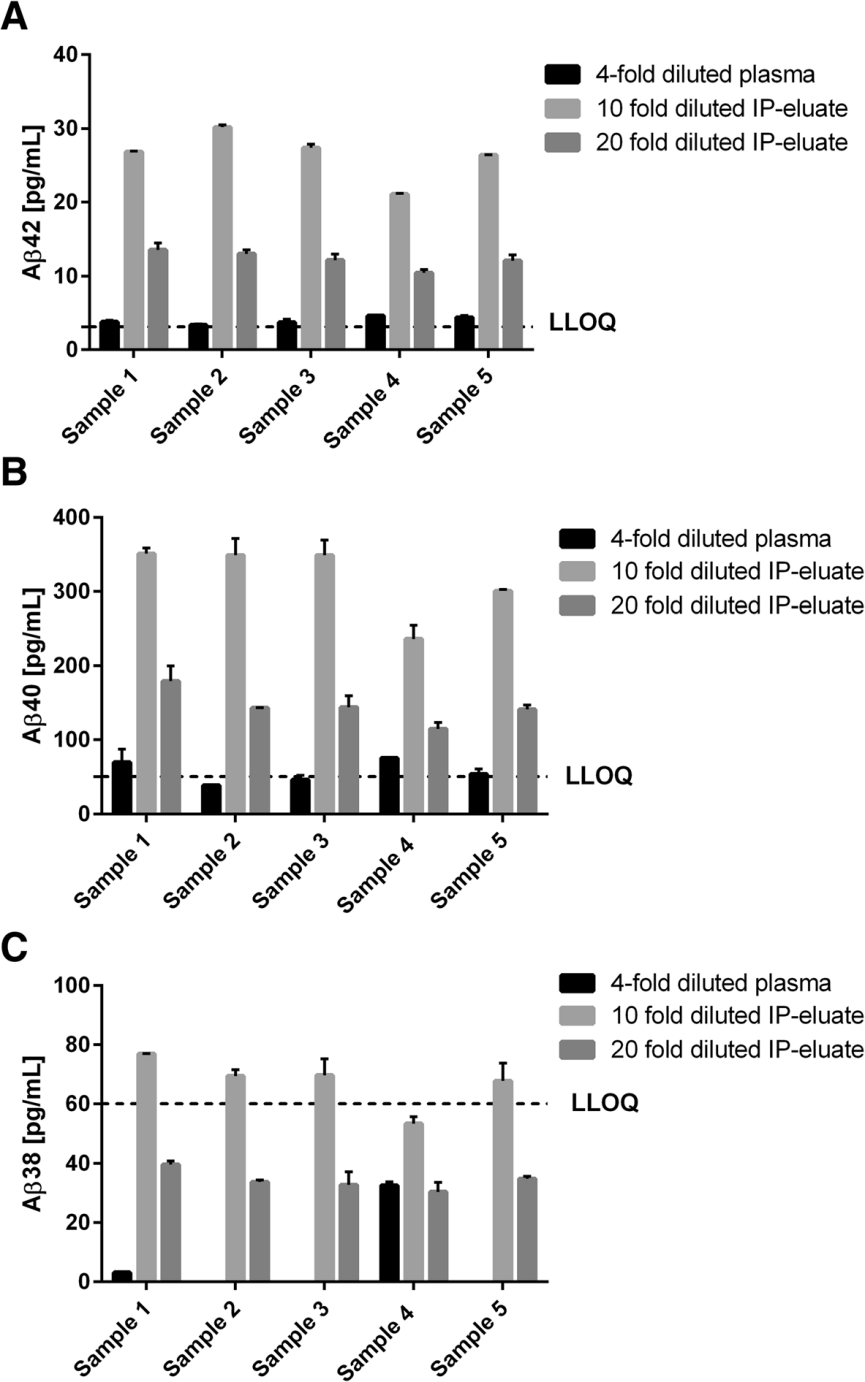
Preconcentration of Aβ peptides from EDTA plasma facilitates semi-quantitative measurement of **a** Aβ42, **b** Aβ40 and **c** Aβ38 in human blood plasma. Bars indicate calculated concentrations in 4-fold diluted EDTA-plasma samples (black bars) and in 10-fold or 20-fold diluted IP eluates (light and dark gray bars) obtained by magnetic bead immunoprecipitation from 800 μl of plasma. Signals above LLOQ of the assay were observed after immunoprecipitation in five out of five tested samples for Aβ42 and Aβ40 and in four out of five samples for Aβ38. Indicated LLOQs were taken from lot-specific certificates of analysis provided with assay kits. Mean ± SD of duplicate reads shown. Aβ amyloid beta, IP immunoprecipitation, LLOQ lower limit of quantification

In contrast, signals clearly above the respective LLOQs for Aβ42 and Aβ40 were observed in five out of five tested samples when the Aβ peptides were preconcentrated by immunoprecipitation from 800 μl of plasma and the resulting IP eluates were measured after 10-fold dilution with Diluent 35. Regarding Aβ38, four out of five samples showed signals above the LLOQ under these conditions. As expected, 20-fold diluted IP eluates produced substantially lower signals than the 10-fold dilutions (Fig. 1). It should be noted that neither the absolute recoveries of the different Aβ peptides after IP nor the true, absolute Aβ concentrations in these samples are known at this stage. Therefore, the measured Aβ concentrations in the plasma IP eluates have to be considered relative Aβ38, Aβ40 and Aβ42 plasma levels.

In the next step, we assessed the variation between parallel IPs. To this end, four different plasma samples were divided into four aliquots of 400 μl each. The resulting 16 aliquots in total were subjected to parallel immunoprecipitations with 25 μl of functionalized magnetic beads per 400 μl of plasma. Each plasma aliquot was treated as an individual sample and underwent the whole IP procedure. The resulting 16 IP eluates (25 μl) were 10-fold diluted, and each one was measured in quadruplicate on one triplex immunoassay plate. For all four original plasma samples investigated, the calculated Aβ peptide concentrations in the eluates from four parallel IPs were in good agreement: The averaged coefficients of variation (%CVs) between parallel immunoprecipitations were 4.3% for Aβ38 (range 2.2–6.3%), 8.7% for Aβ40 (range 2.2–15.5%) and 6.1% for Aβ42 (range 2.1–8.3%) (Table 2).

**Table 2:** Variation of calculated Aβ concentrations^a^ between four parallel immunoprecipitations

	Aβ38 (pg/ml)			Aβ40 (pg/ml)			Aβ42 (pg/ml)		
	Mean	SD	%CV	Mean	SD	%CV	Mean	SD	%CV
Sample 1	54.08	2.38	4.40	207.22	32.06	15.47	20.38	1.45	7.12
Sample 2	54.04	3.39	6.27	216.78	26.08	12.03	23.45	1.94	8.26
Sample 3	55.44	1.19	2.15	201.50	4.50	2.23	20.12	0.43	2.12
Sample 4	46.37	2.01	4.34	162.46	8.15	5.01	18.60	1.29	6.93
Overall mean	52.48	2.24	4.29	196.99	17.70	8.69	20.64	1.28	6.11

*Aβ* amyloid beta, *%CV* coefficient of variation, *SD* standard deviation

^a^Indicated mean concentrations refer to averaged calculated concentrations (pg/ml) in diluted immunoprecipitation eluates without correction for 10-fold dilution before measurement with the Aβ triplex immunoassay

The coefficients of variation between technical replicates on the same immunoassay plate in this experiment (see earlier) were 9.7% ± 5.5 (mean ± SD) for Aβ40, 7.8% ± 5.1 for Aβ42 and 7.9% ± 2.5 for Aβ38.

### Assessment of plasma Aβ38, Aβ40 and Aβ42 in patients with dementia of the Alzheimer type and dementia due to other reasons

Next, we applied an optimized version of the two-step immunoassay protocol to the measurement of the relative levels of Aβ38, Aβ40 and Aβ42 in EDTA plasma in a clinical cohort comprising 40 dementia patients. All 40 tested samples showed Aβ40 and Aβ42 signals in the quantitative range of the respective assays. The Aβ38 signals were in the quantitative range of the assay in 36 samples, while the remaining four (10%) were in the detectable range but below the quantitative range of the assay. The standard curves for the three Aβ peptides, the assay detection ranges and the Aβ levels in the IP eluates are displayed in Additional files 1, 2 and 3.

Each IP eluate was analyzed in four technical replicates. The mean %CVs between the four technical replicates (intra-assay variation) of the calculated Aβ concentrations measured in the 40 clinical samples were 4.0% (range 0.4–9%) for Aβ38, 4.0% (range 1.1–7.7%) for Aβ40 and 4.4% (range 1.0–10.3%) for Aβ42 (see also Additional file 4).

The calculated Aβ40 and Aβ42 concentrations in the diluted IP eluates were not normally distributed. Instead, a Gaussian distribution was observed after log transformation (see Additional file 5). Therefore, further statistical analysis was done on log2-transformed data.

**Fig. 2:**
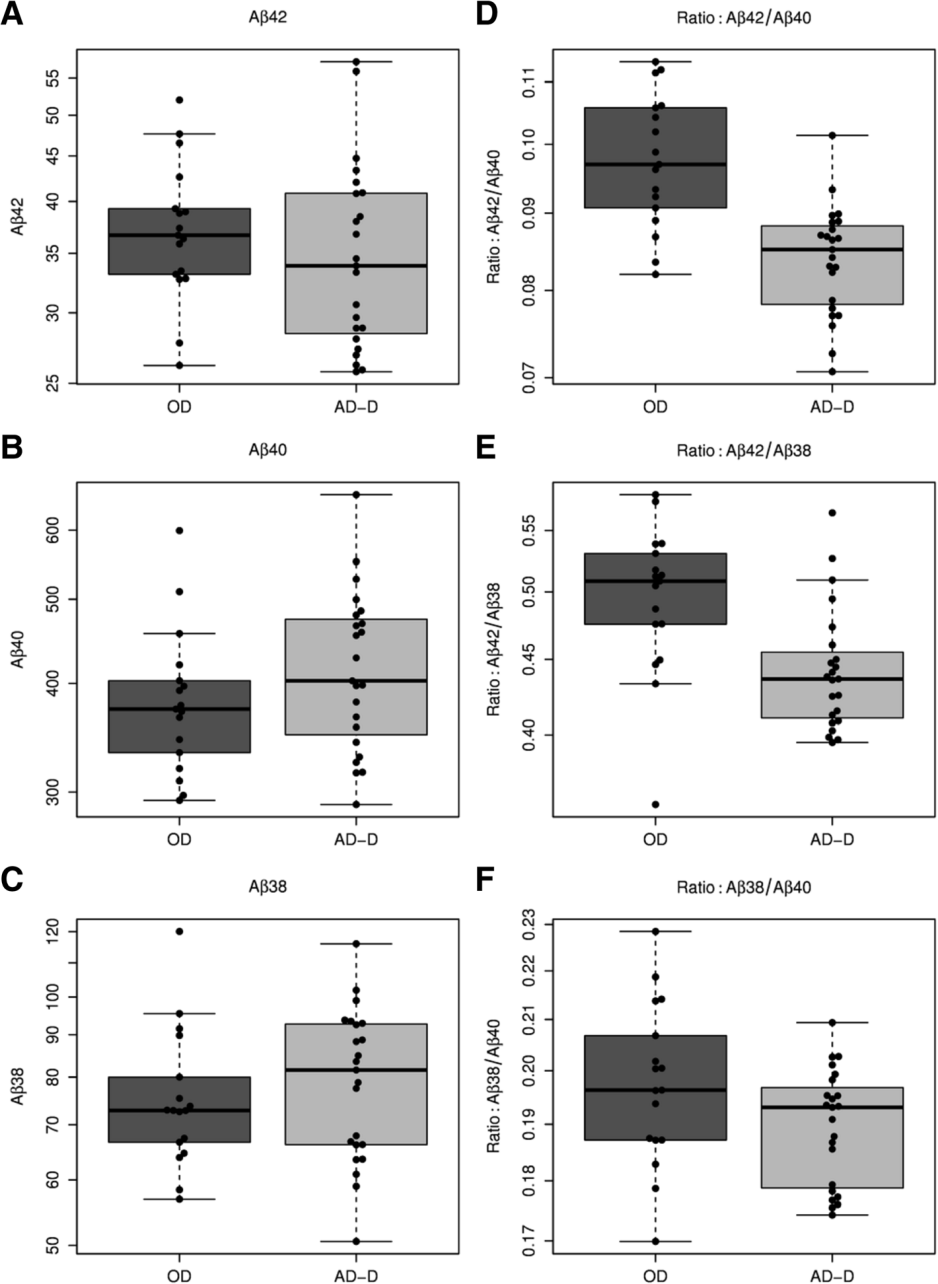
Dichotomized comparison of levels of Aβ38, Aβ40 and Aβ42 and concentration ratios Aβ42/Aβ40, Aβ42/Aβ38 and Aβ38/Aβ40 in diluted IP eluates. No statistically significant differences in levels of **a** Aβ42, **b** Aβ40 and **c** Aβ38 between diagnostic groups. In contrast, significantly lower **d** Aβ42/Aβ40 (*t* test *p* < 0.001) and **e** Aβ42/Aβ38 (*t* test *p* = 0.0031) ratios observed in AD-D group compared to OD group. **f** Aβ38/Aβ40 ratio did not differ significantly between diagnostic groups (*p* = 0.0785). Statistical tests were done after log transformation. Accordingly, scaling of *y* axes is logarithmic. Aβ amyloid beta, AD-D dementia of the Alzheimer type, OD dementia due to other reasons

To compare the relative plasma levels of the three Aβ peptides between groups, we dichotomized the clinical sample into the categories AD-D and OD. Interestingly, there were no statistically significant group differences regarding the plasma levels of the single Aβ peptides Aβ38, Aβ40 or Aβ42 in the IP eluates. In sharp contrast, the concentration ratios Aβ42/40 and Aβ42/38 were significantly lower in the AD-D group compared to the OD patients (Fig. 2 and Additional file 6).

### Receiver-operating characteristic analysis

**Fig. 3:**
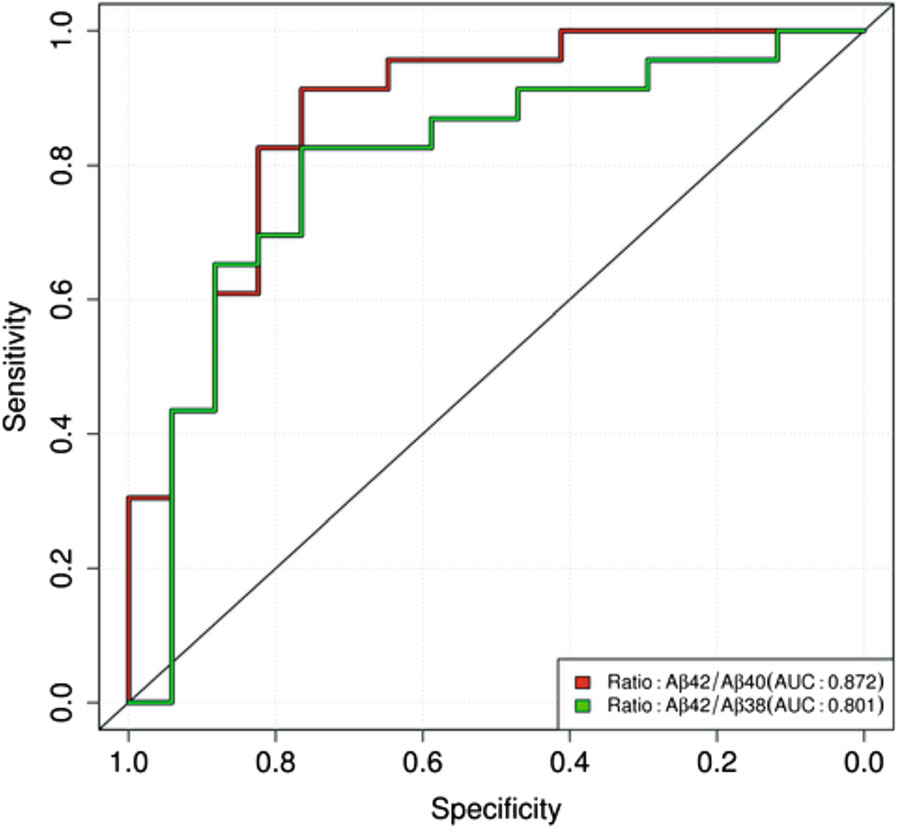
Biomarker performance of Aβ42/Aβ40 and Aβ42/Aβ38 ratios in plasma. ROC analyses for Aβ42/Aβ40 and Aβ42/Aβ38 ratios for classification of OD vs AD-D. For both ROC curves, null hypothesis that AUCs were equal to 0.5 could be rejected (*p* < 0.001 for Aβ42/Aβ40 ratio and *p* = 0.0013 for Aβ42/Aβ38 ratio). Aβ amyloid beta, AUC area under the curve

The performance of the six blood variables Aβ38, Aβ40, Aβ42, Aβ42/Aβ40 ratio, Aβ42/Aβ38 ratio and Aβ38/Aβ40 ratio as potential AD biomarkers was evaluated by ROC analysis. For the classification, we compared the diagnostic groups OD vs AD-D and calculated single-value ROC curves. The null hypothesis that the area under the curve (AUC) was equal to 0.5 was tested for each ROC curve with the GraphPad Prism software (see earlier). Significant *p* values were obtained for the ROC curves for the Aβ42/Aβ40 ratio (*p* < 0.001) and the Aβ42/Aβ38 ratio (*p* = 0.0013) (Fig. 3), but not for any of the other tested variables. The results of the ROC analyses are summarized in Additional file 7. The AUCs were 0.872 for the Aβ42/Aβ40 ratio and 0.801 for the Aβ42/Aβ38 ratio. Both ROC curves were not significantly different from each other (*p* = 0.071). This was not unexpected since the levels of Aβ38 and Aβ40 in the IP eluates were strongly correlated with each other (Pearson *r* = 0.944, *p* < 0.001; see Additional file 8).

### Correlations between the Aβ variables in plasma and CSF biomarkers

**Fig. 4:**
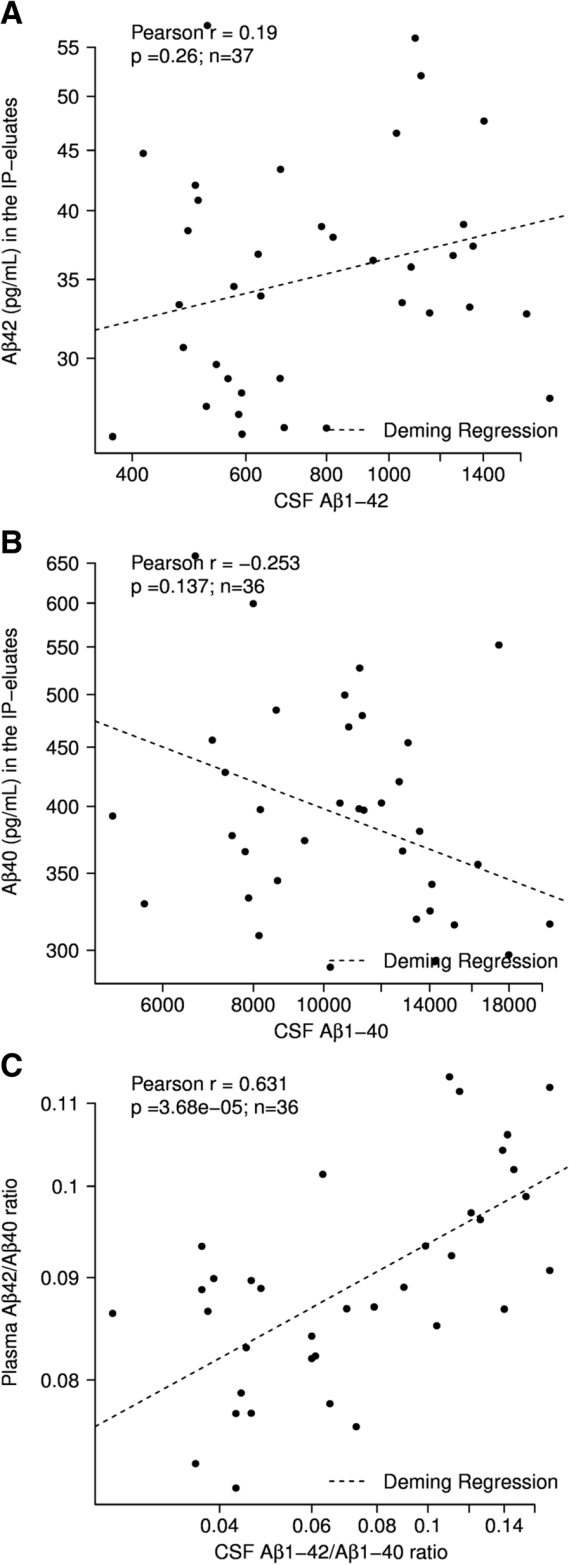
Correlations between Aβ measures in CSF and plasma. No statistically significant correlations observed between **a** CSF Aβ1–42 and plasma Aβ42 or **b** CSF Aβ1–40 and plasma Aβ40. **c** Aβ1–42/Aβ1–40 ratio in CSF statistically significantly correlated with plasma Aβ42/Aβ40 ratio. Scatterplots of indicated Aβ variables in blood plasma and CSF are shown. Pearson correlation coefficients annotated and Deming regressions shown as dashed lines. Statistical tests were done after log transformation. Accordingly, scaling of *x* and *y* axes is logarithmic. Aβ amyloid beta, CSF cerebrospinal fluid, IP immunoprecipitation

For the majority of the study participants, Aβ1–42 (*n* = 37) and Aβ1–40 (*n* = 36) concentrations in CSF determined in a clinical routine laboratory for diagnostic reasons were available. No statistically significant correlations were observed between plasma Aβ40 and CSF Aβ1–40 (Pearson *r* = 0.19, *p* = 0.26) or plasma Aβ42 and CSF Aβ1–42 (Pearson *r* = − 0.253, *p* = 0.137). In contrast, the Aβ1–42/Aβ1–40 ratio in CSF and the plasma Aβ42/Aβ40 ratio were statistically highly significantly associated (Pearson *r* = 0.631, *p* < 0.001 (Fig. 4). In addition, we observed significant correlations between the plasma Aβ42/Aβ40 ratio and CSF tTau (*r* = − 0.579, *p* < 0.001), CSF pTau181 (*r* = − 0.439, *p* = 0.0064) and the ratio Aβ1–42/tTau (*r* = 0.694, *p* < 0.001). In those 16 study subjects for whom quantifiable amyloid-PET data were available, we observed a statistically significant correlation between the amyloid-PET SUVr data and the plasma Aβ42/Aβ40 ratio (Pearson *r* = − 0.745, *p* < 0.001). A multiple testing correction was not applied.

### Magnitude of the differences between the groups

The observed plasma Aβ42/Aβ40 ratios in the AD-D and OD groups were 0.084 ± 0.007 (mean ± SD) and 0.098 ± 0.01, respectively (Additional file 6). In CSF, the mean Aβ1–42/Aβ1–40 ratios were 0.052 ± 0.018 in the AD group (*n* = 21) and 0.126 ± 0.030 in the OD group (*n* = 15) (Table 1). Hence, in the AD-D patients the mean plasma Aβ42/Aβ40 ratio was decreased by 14% whereas in CSF the mean Aβ1–42/Aβ1–40 ratio was 58% lower in AD-D patients than in the OD group.

### The effects of *APOE* ε4 and gender

**Fig. 5:**
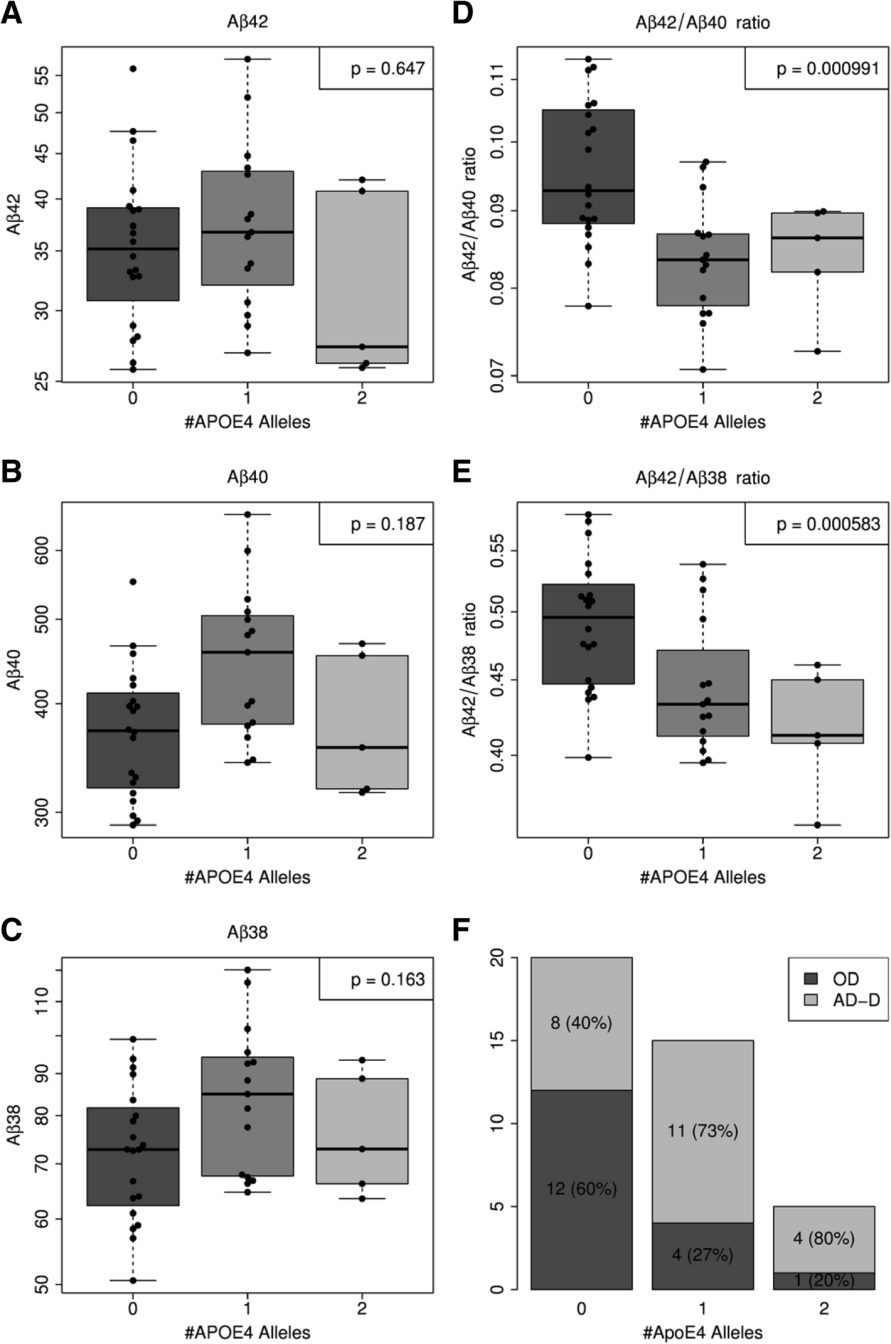
Effect of *APOE* ε4 on Aβ measures in IP eluates and on frequency of dichotomized diagnostic classifications*.* No statistically significant effects of number of *APOE* ε4 alleles on plasma levels of **a** Aβ42, **b** Aβ40 and **c** Aβ38. In contrast, *APOE* genotype had a statistically significant effect on **d** Aβ42/Aβ40 (*F* test *p* < 0.001) and **e** Aβ42/Aβ38 (*F* test *p* < 0.001) ratios. **f** Proportion of AD-D patients seemed to increase with increasing number of *APOE* ε4 alleles. However, in the small sample size studied, this effect did not reach statistical significance (Fisher *p* value = 0.1). Statistical test were done after log transformation. Accordingly, scaling of *y* axes is logarithmic in (a)–(e). Aβ amyloid beta, AD-D dementia of the Alzheimer type, APOE apolipoprotein E, OD dementia due to other reasons

We observed statistically significant effects of the *APOE* genotype (zero, one or two *APOE* ε4 alleles) on the Aβ42/Aβ40 and the Aβ42/Aβ38 ratios but not on any of the three Aβ peptides analyzed per se (Fig. 5). Those individuals with one or two *APOE* ε4 alleles showed lower Aβ42/Aβ40 ratios (*F* test *p* < 0.001) and lower Aβ42/Aβ38 ratios (*F* test *p* < 0.001) than those without *APOE* ε4 (Fig. 5d, e). Based on the dichotomized diagnostic classification (OD vs AD-D), the proportion of OD patients appeared to decrease with an increasing number of *APOE* ε4 alleles (Fig. 5f). However, in the small sample size that was studied, this effect did not reach statistical significance (Fisher *p* value = 0.1). There was no statistically significant relation between gender and diagnosis according to Fisher’s exact test (*p* = 0.116). Furthermore, we did not observe any significant gender effects on the Aβ levels or ratios in the plasma IP eluates. The observed effects were similar when a multivariate regression model with all three covariates (*APOE*, age and gender) was used.

### Reproducibility of the IP-MSD measurements between different runs

To assess the interassay reproducibility of the two-step immunoassay, the experiment was repeated in the same set of clinical samples 4 months after the initial test. The protocol was slightly modified, for example to adjust for a different starting volume of plasma and Aβ measurements in two technical replicates instead of four. The mean %CVs between the two different two-step immunoassay runs were 10.2% ± 6.05 for Aβ38 (range 0.15–20.48%), 6.4% ± 4.33 for Aβ40 (range 0.27–16.52%), 8.1% ± 6.66 for Aβ42 (range 0.12–27.15%) and 8.3% ± 4.8 for the Aβ42/Aβ40 ratio (range 0.4–19.38%) (Table 3). However, the Bland–Altman plot suggested nonrandom differences between the two experiments regarding Aβ38, Aβ42, the Aβ42/Aβ40 ratio and the Aβ42/Aβ38 ratio (see Additional file 9).

**Table 3:** Reproducibility between two different experiments

	%CV mean	%CV SD	%CV minimum	%CV maximum
Aβ38	10.2	6.05	0.15	20.48
Aβ40	6.42	4.33	0.27	16.52
Aβ42	8.11	6.66	0.12	27.15
Aβ42/Aβ40 ratio	8.28	4.80	0.40	19.38

*Aβ* amyloid beta, *%CV* coefficient of variation, *SD* standard deviation

## Discussion

We report on the development of a prototypic novel two-step immunoassay for the simultaneous assessment of the relative concentrations of Aβ38, Aβ40 and Aβ42 in human EDTA blood plasma. The reliable measurement of Aβ peptides in blood is hampered by very low Aβ concentrations and high levels of particular blood proteins and other compounds which may interfere with the immunological detection and quantification of Aβ. Two very recent studies provided strong evidence that the Aβ42/Aβ40 ratio [6], Aβ1–40/1–42 ratio and APP669–711/Aβ1–42 ratio [7] in plasma may represent highly attractive AD biomarker candidates that can predict cerebral amyloid pathology with very high accuracy. In both studies, plasma Aβ peptides were first preconcentrated by immunoprecipitation followed by quantification by mass spectrometry. For us, an obvious question arising from these reports was whether Aβ immunoprecipitation might also be able to facilitate the measurement of plasma Aβ by high-throughput-capable, classical immunoassays. For pre-enrichment of Aβ peptides from blood plasma, we employed the monoclonal antibody 1E8 covalently coupled to magnetic beads. The antibody recognizes an amino-terminal epitope within the Aβ sequence [17] and was previously used by our group for immunoprecipitating Aβ peptides from plasma for subsequent analysis by one or two-dimensional urea SDS polyacrylamide gel-electrophoresis followed by western blot analysis [9, 18]. To reduce nonspecific binding and to release plasma Aβ peptides from potential other binding partners in blood, the immunoprecipitation was performed in the presence of three detergents, namely SDS, sodium desoxycholate and Igepal CA-630 (Nonidet P40). Elution of the captured Aβ peptides from the washed magnetic bead immune complexes was achieved by heating in a small volume of bicine-CHAPS buffer, which allows for selective elution of Aβ with negligible bleeding of sAPPα or IgG [14]. CHAPS is a nondenaturing zwitterionic detergent [19], and the bicine-CHAPS elution buffer has a pH value of 7.6. Thus, the IP eluates are compatible with subsequent immunoassays without the need for pH neutralization or removal of denaturing substances. For the simultaneous measurement of Aβ38, Aβ40 and Aβ42 in the IP eluates, we employed a commercially available chemiluminescence multiplex immunoassay, which we have previously validated for measuring all three Aβ variants in CSF [15].

The observations from our current study indicate that the pre-enrichment of plasma Aβ peptides by magnetic bead IP is compatible with the multiplex immunoassay and allows for the semi-quantitative simultaneous measurement of Aβ40, Aβ42 and Aβ38 in the IP eluates. The observed technical intra-assay variation between parallel manual immunoprecipitations and between technical replicate reads of diluted IP eluates on the same assay plate was acceptable. The comparison of two independent runs of the novel two-step immunoassay with the same set of 40 plasma samples revealed interassay coefficients of variation ranging from 0.12 to 27.2% with mean CV values of 10.2% ± 6.1 (Aβ38), 6.4% ± 4.3 (Aβ40) and 8.1% ± 6.7 (Aβ42) (Table 2). The observed degree of interassay variation appears to be comparable with previous findings regarding the assessment of diluted CSF with the same chemiluminescence Aβ multiplex assay kit [15]. However, Bland–Altman plots suggested nonrandom errors between the two different experiments regarding the measurements of Aβ38 and Aβ42. The observations regarding interassay reproducibility have to be considered preliminary, and additional experiments including reference samples will be needed for a more comprehensive assessment. Critical points to be addressed in future studies include the use of internal control samples for normalization between different assay plates and experiments, standardization of preanalytical and analytical procedures, extended assessment of the interassay reproducibility and automatization of manual steps, in particular the Aβ pre-enrichment by IP.

In the current pilot study, we have assessed a small clinical cohort, which included 15 patients with probable AD dementia, eight patients with possible AD dementia and 17 patients with dementia due to other reasons. For the dichotomized statistical evaluation, we combined the patients with probable and possible AD dementia into the single category AD-D (dementia of the Alzheimer’s type). Both probable and possible AD patients showed CSF-biomarker or imaging evidence for cerebral amyloid pathology. In all of the plasma samples analyzed, the measured chemiluminescent signals in the IP eluates were in the quantitative range of the assay for Aβ40 and Aβ42 and in 90% of the cases for Aβ38. There were no statistically significant differences in the plasma levels of any of the three Aβ peptides between the diagnostic groups OD and AD-D. However, the Aβ42/Aβ40 ratio and the Aβ42/Aβ38 ratio were statistically significantly decreased in the AD-D group. ROC analyses for the discrimination between OD and AD-D yielded an AUC of 0.87 for the Aβ42/Aβ40 ratio and 0.80 for the Aβ42/Aβ38 ratio. The plasma levels of Aβ38 and Aβ40 were strongly correlated, and thus it is not clear at this stage whether measuring Aβ38 in plasma in a larger cohort may provide an added value for differential dementia diagnosis. However, in CSF low levels of Aβ1–38 or Aβ38, respectively, were observed in patients with frontotemporal dementia [20, 21]. Furthermore, the CSF Aβ42/Aβ38 ratio may represent a biomarker candidate to support the discrimination between AD and dementia with Lewy bodies [22].

The mean Aβ42/40 ratio in the plasma IP eluates in our sample was approximately 14% lower in the AD-D group than in the OD patients. In contrast, in the same clinical cohort, the mean CSF Aβ1–42/Aβ1–40 ratio in the AD-D group was reduced by approximately 58%. The observations from IP-MS studies [6, 7, 23] suggest that the actual AD biomarker is presumably a decrease in soluble Aβ1–42 in the central nervous system that can also be detected in blood plasma. Aβ40, Aβ1–40 or APP669/711, which are apparently not altered in AD patients, can serve as an internal control harmonizing large differences in absolute Aβ peptide blood concentrations, which is essential to improve the discrimination between diagnostic groups. The smaller relative decrease in the Aβ42/Aβ40 ratio in AD-D in plasma than in CSF we report here can be explained by dilution due to a major fraction of Aβ peptides in blood that is not brain derived but stems from peripheral sources [6, 24, 25].

Our findings are in agreement with some previous reports suggesting that a low Aβ42/Aβ40 ratio and/or a low Aβ42 level in plasma represent candidate biomarkers of neuropathological changes related to AD [6, 11, 24, 26–29]. However, this is not supported by several other studies [25, 30–32]. Consequently, two different comprehensive meta-analyses did not reveal strong associations of plasma Aβ peptides with AD [1, 33]. We assume that the conflicting study results can be explained, at least in part, by very low Aβ concentrations in blood, binding of Aβ to other proteins, the presence of substances that can interfere with the immunological detection and different assay formats or antibodies that were employed. It appears that amino-terminal-selective immunoprecipitation in the presence of detergents represents a valuable strategy to minimize epitope masking and matrix effects, and can thus facilitate the reliable measurement of plasma Aβ peptides. Further studies are needed for technical advancement of our novel, prototypic two-step immunoassay such as an automated high-throughput-capable assay format. Moreover, the preliminary findings regarding the biomarker performance of the plasma Aβ42/Aβ40 and Aβ42/Aβ38 ratios we have determined in this pilot study need to be validated in independent larger patient cohorts.

## Conclusions

Taken together, the observations from this study suggest that pre-enrichment of Aβ peptides from blood plasma by immunoprecipitation with an antibody against the Aβ N-terminus and in the presence of detergents can substantially facilitate the measurement of specific Aβ variants by a commercially available multiplex immunoassay. Furthermore, our findings support the results of two recent IP-MS studies reporting highly accurate prediction of cerebral amyloid pathology by the blood plasma ratios Aβ42/Aβ40 [6] and Aβ1–40/Aβ1–42 [7]. Major limitations of our current study include the small sample size, the need for assay standardization and automatization of manual steps, and the need for inclusion of reference material for normalization between assay plates and experiments. These points will have to be addressed in continuative future studies.

## Additional files

Additional file 1: Aβ38 standard curves of the two MSD assay plates applied to analysis of the clinical sample. (PDF 258 kb)

Additional file 2: Aβ40 standard curves of the two MSD assay plates applied to analysis of the clinical sample. (PDF 283 kb)

Additional file 3: Aβ42 standard curves of the two MSD assay plates applied to analysis of the clinical sample. (PDF 277 kb)

Additional file 4: Intra-assay variation of the multiplex Aβ immunoassay. (PDF 51 kb)

Additional file 5: Baseline statistics of measured Aβ38, Aβ40 and Aβ42 levels in diluted IP eluates and the concentration ratios Aβ42/Aβ40, Aβ42/Aβ38 and Aβ38/Aβ40. (PDF 183 kb)

Additional file 6: Comparison of measured Aβ levels and Aβ ratios in IP eluates from plasma between diagnostic groups. (PDF 182 kb)

Additional file 7: Summary of ROC analysis results. (PDF 184 kb)

Additional file 8: Concentrations of Aβ38 and Aβ40 in IP eluates were strongly correlated with each other. (PDF 96 kb)

Additional file 9: Bland–Altman plots suggesting systematic variations between two independent two-step immunoassay experiments. (PDF 99 kb)

## Abbreviations

%CV: Coefficient of variation
^18^F-FDG-PET: ^18^F-fluorodeoxyglucose positron emission tomography
AD: Alzheimer’s disease
AD-D: Dementia of the Alzheimer type
Amyloid-PET: Amyloid-beta positron emission tomography
*APOE*: Apolipoprotein E
Aβ: Amyloid beta
cCT: Cranial computed tomography
CHAPS: 3-((3-Cholamidopropyl)dimethylammonio)-1-propanesulfonate
CSF: Cerebrospinal fluid
IP: Immunoprecipitation
LLOD: Lower limit of detection
LLOQ: Lower limit of quantification
MRI: Magnetic resonance imaging
MS: Mass spectrometry
OD: Dementia due to other reasons
PCR: Polymerase chain reaction
pTau: Phosphorylated Tau protein
ROC: Receiver operating characteristic
SUV: Standardized uptake value
SUVr: Standardized uptake value ratio
tTau: Total Tau protein
