# Supplementary material for: A two-step immunoassay for the simultaneous assessment of Aβ38, Aβ40 and Aβ42 in human blood plasma supports the Aβ42/Aβ40 ratio as a promising biomarker candidate of Alzheimer’s disease
Source: Alzheimers Res Ther. 2018 Dec 8;10:121. doi: 10.1186/s13195-018-0448-x (PMC6286509; doi:10.1186/s13195-018-0448-x)
Supplement: Supplementary file 4 — Intra-assay variation of the multiplex Aβ immunoassay. (PDF 51 kb) [file 13195_2018_448_MOESM4_ESM.pdf]

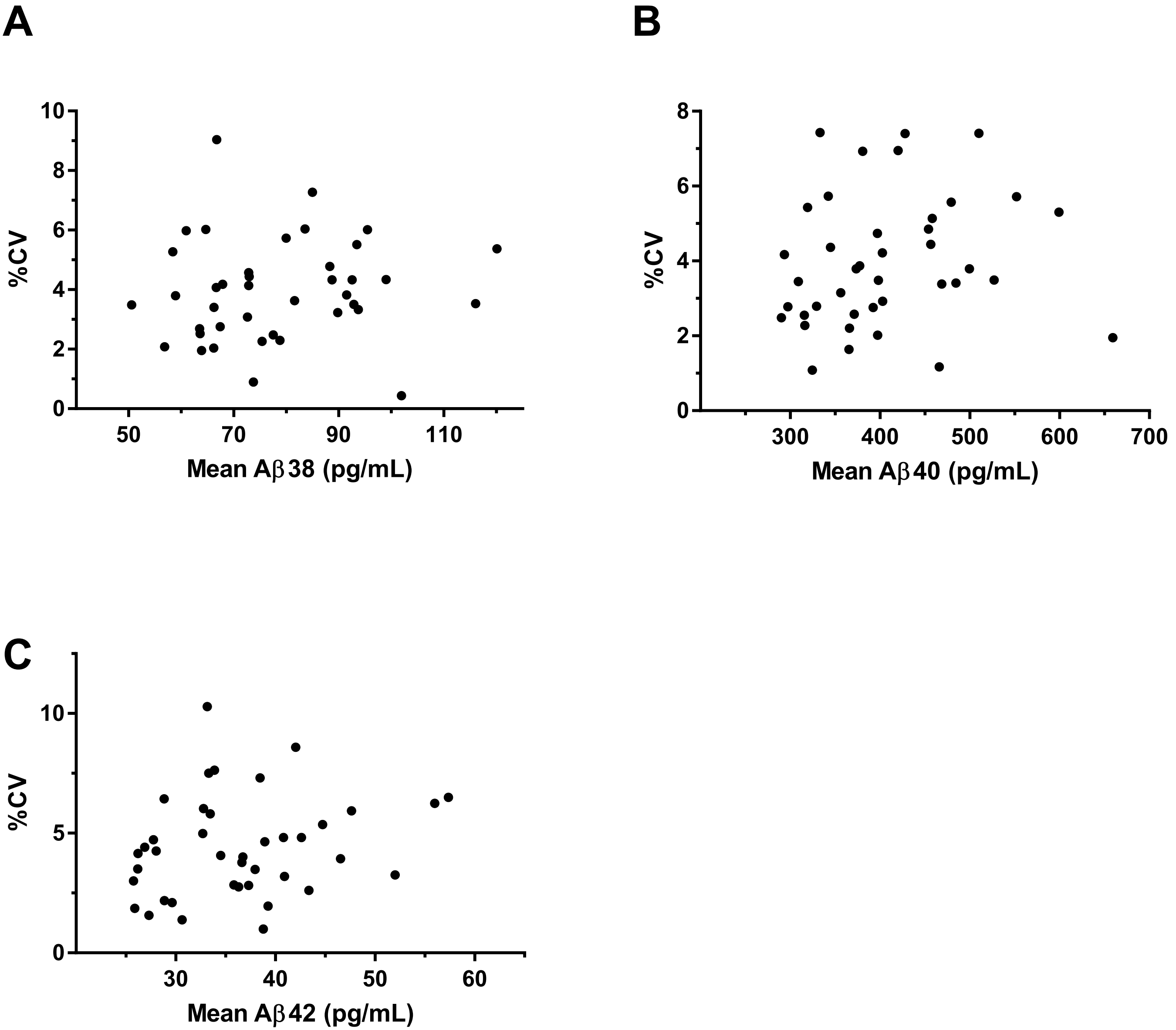

Additional file 4. Intra-assay variation of the MSD multiplex Aβ immunoassay. Diluted IP-eluates (n=40) were analyzed with the MSD multiplex immunoassay in 4 replicates measured in parallel. The coefficients of variation (%CVs) between the technical replicates are plotted against the average concentration of A) Aβ38, B) Aβ40 and C) Aβ42. We did not observe statistically significant correlations between the average concentrations and CVs of the quadruplicate measurements for any of the tested Aβ peptides (Aβ38: Pearson $r = 0.081$, $p = 0.62$; Aβ40: Pearson $r = 0.177$, $p = 0.27$, Aβ42: Pearson $r = 0.22$, $p = 0.17$).
