# Supplementary material for: A two-step immunoassay for the simultaneous assessment of Aβ38, Aβ40 and Aβ42 in human blood plasma supports the Aβ42/Aβ40 ratio as a promising biomarker candidate of Alzheimer’s disease
Source: Alzheimers Res Ther. 2018 Dec 8;10:121. doi: 10.1186/s13195-018-0448-x (PMC6286509; doi:10.1186/s13195-018-0448-x)
Supplement: Supplementary file 5 — Baseline statistics of measured Aβ38, Aβ40 and Aβ42 levels in diluted IP eluates and the concentration ratios Aβ42/Aβ40, Aβ42/Aβ38 and Aβ38/Aβ40. (PDF 183 kb) [file 13195_2018_448_MOESM5_ESM.pdf]

Additional file 5:

Baseline statistics of the measured Aβ38, Aβ40 and Aβ42 levels in the diluted IP-eluates and the concentration ratios Aβ42/Aβ40, Aβ42/Aβ38 and Aβ38/Aβ40

	N	Min	1st Quantile	Median	Mean	3rd Quantile	Max	SD	P-value normality test	P-value normality test (log2)
Aβ42	40	25.76	29.44	36.08	36.40	40.84	57.37	8.10	0.027	0.232
Aβ40	40	290.22	340.00	394.73	406.47	460.06	659.34	86.40	0.025	0.358
Aβ38	40	50.57	66.23	74.56	78.31	90.23	120.09	16.02	0.117	0.681
Aβ42/Aβ40	40	0.07	0.08	0.09	0.09	0.10	0.11	0.01	0.167	0.500
Aβ42/Aβ38	40	0.36	0.43	0.45	0.47	0.51	0.58	0.06	0.251	0.453
Aβ38/Aβ40	40	0.17	0.18	0.19	0.19	0.20	0.23	0.01	0.445	0.655
