# Supplementary material for: A two-step immunoassay for the simultaneous assessment of Aβ38, Aβ40 and Aβ42 in human blood plasma supports the Aβ42/Aβ40 ratio as a promising biomarker candidate of Alzheimer’s disease
Source: Alzheimers Res Ther. 2018 Dec 8;10:121. doi: 10.1186/s13195-018-0448-x (PMC6286509; doi:10.1186/s13195-018-0448-x)
Supplement: Supplementary file 6 — Comparison of measured Aβ levels and Aβ ratios in IP eluates from plasma between diagnostic groups. (PDF 182 kb) [file 13195_2018_448_MOESM6_ESM.pdf]

Additional file 6

Comparison of the measured Aβ levels and Aβ-ratios in IP-eluates from plasma between the diagnostic groups

	AD-D			OD			
	Mean	Median	SD	Mean	Median	SD	P-value*
Aβ42 (pg/mL)	35.6	33.9	9.0	37.5	36.6	6.8	0.321
Aβ40 (pg/mL)	421.6	402.7	90.2	386.0	373.6	78.9	0.189
Aβ38 (pg/mL)	79.9	81.6	16.4	76.2	72.9	15.7	0.486
Aβ42/Aβ40	0.084	0.085	0.007	0.098	0.097	0.010	2.37E-05
Aβ42/Aβ38	0.444	0.436	0.044	0.497	0.508	0.055	0.0031
Aβ38/Aβ40	0.190	0.193	0.010	0.198	0.196	0.015	0.0785

*unpaired t-test for differences between the two diagnostic categories.
