# Supplementary material for: A two-step immunoassay for the simultaneous assessment of Aβ38, Aβ40 and Aβ42 in human blood plasma supports the Aβ42/Aβ40 ratio as a promising biomarker candidate of Alzheimer’s disease
Source: Alzheimers Res Ther. 2018 Dec 8;10:121. doi: 10.1186/s13195-018-0448-x (PMC6286509; doi:10.1186/s13195-018-0448-x)
Supplement: Supplementary file 7 — Summary of ROC analysis results. (PDF 184 kb) [file 13195_2018_448_MOESM7_ESM.pdf]

Additional file 7

Summary of ROC analysis results

Parameter tested	Aβ38	Aβ40	Aβ42	ratio Aβ42/40	ratio Aβ42/38	ratio Aβ38/40
AUC	0.573	0.627	0.591	0.872	0.801	0.661
Standard error*	0.093	0.090	0.091	0.058	0.076	0.091
95% Confidence interval	0.390 to 0.756	0.450 to 0.804	0.413 to 0.769	0.759 to 0.986	0.651 to 0.950	0.484 to 0.839
P value	0.436	0.176	0.331	<0.0001	0.0013	0.085
Threshold	76.44	397.26	31.68	0.09	0.474	0.196
Specificity	0.706	0.706	0.882	0.765	0.765	0.588
Sensitivity	0.609	0.609	0.435	0.913	0.826	0.739
Accuracy	0.650	0.650	0.625	0.85	0.800	0.675
True positive	14	14	10	21	19	17
False positive	5	5	2	4	4	7
False negative	9	9	13	2	4	6
True negative	12	12	15	13	13	10
Negative predictive value	0.571	0.571	0.536	0.867	0.765	0.625
Positive predictive value	0.737	0.737	0.833	0.840	0.826	0.708

*The standard error of the AUC is calculated by the Prism software according to Hanley JA, McNeil BJ, Radiology 1982, 143 29-36 (cited in: URL: https://www.graphpad.com/guides/prism/6/statistics, accessed: 2018-10-08).
