# Supplementary material for: A two-step immunoassay for the simultaneous assessment of Aβ38, Aβ40 and Aβ42 in human blood plasma supports the Aβ42/Aβ40 ratio as a promising biomarker candidate of Alzheimer’s disease
Source: Alzheimers Res Ther. 2018 Dec 8;10:121. doi: 10.1186/s13195-018-0448-x (PMC6286509; doi:10.1186/s13195-018-0448-x)
Supplement: Supplementary file 8 — Concentrations of Aβ38 and Aβ40 in IP eluates were strongly correlated with each other. (PDF 96 kb) [file 13195_2018_448_MOESM8_ESM.pdf]

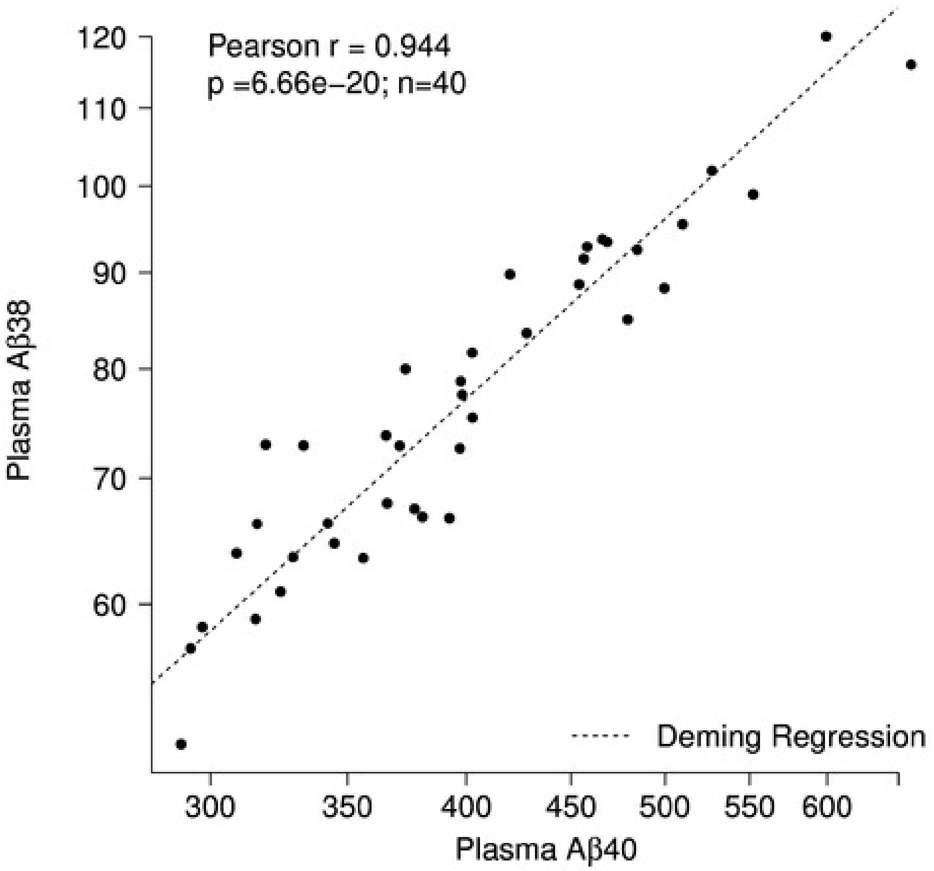

Additional file 8: The concentrations of Aβ38 and Aβ40 in the IP-eluates were strongly correlated with each other.
