# Supplementary material for: A two-step immunoassay for the simultaneous assessment of Aβ38, Aβ40 and Aβ42 in human blood plasma supports the Aβ42/Aβ40 ratio as a promising biomarker candidate of Alzheimer’s disease
Source: Alzheimers Res Ther. 2018 Dec 8;10:121. doi: 10.1186/s13195-018-0448-x (PMC6286509; doi:10.1186/s13195-018-0448-x)
Supplement: Supplementary file 9 — Bland–Altman plots suggesting systematic variations between two independent two-step immunoassay experiments. (PDF 99 kb) [file 13195_2018_448_MOESM9_ESM.pdf]

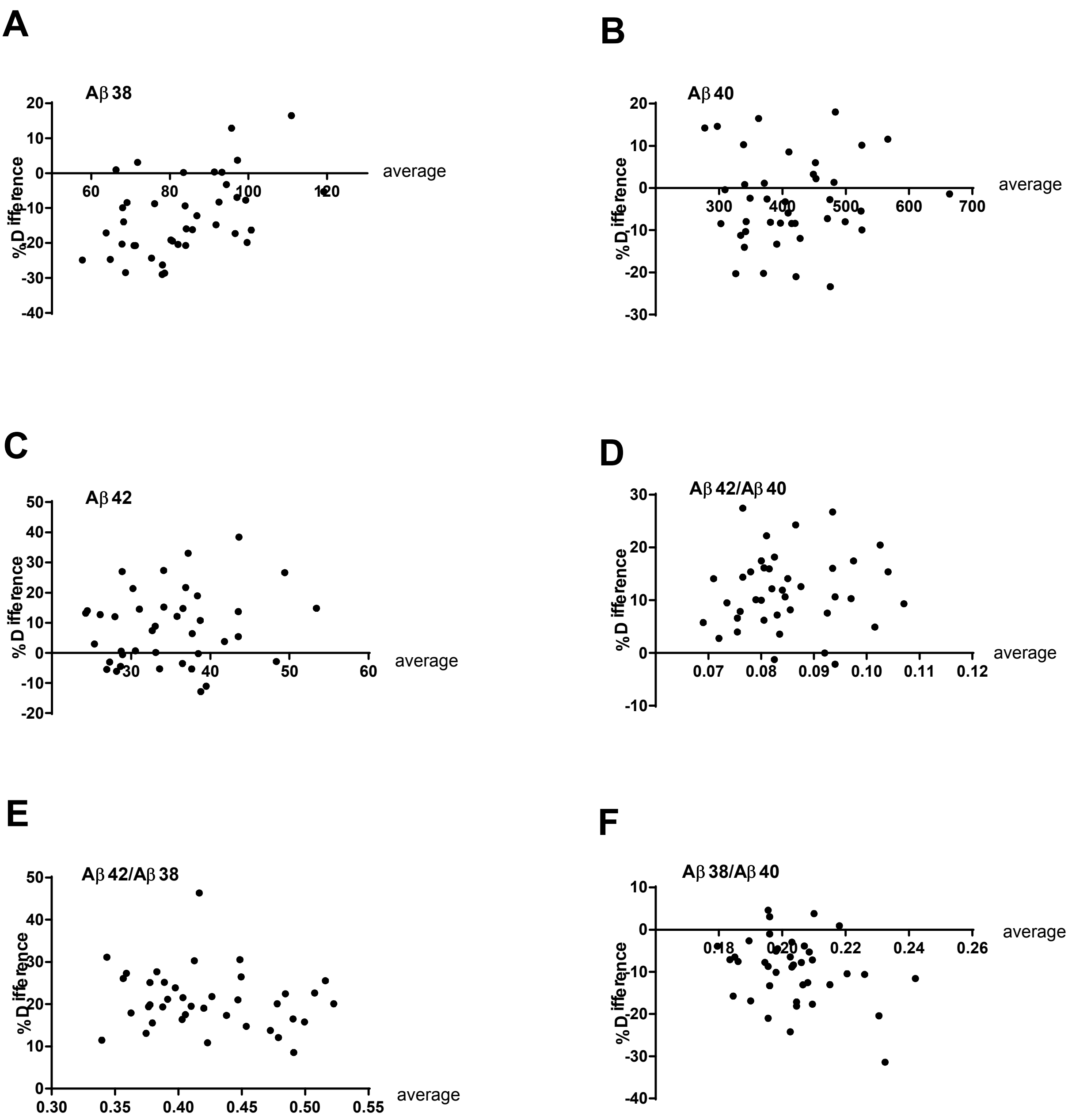

Additional file 9: Bland-Altman plots suggest systematic variations between two independent two-step immunoassay experiments. The relative differences between two experiments in the measured levels of A) Aβ38, B) Aβ40, C) Aβ42 and the ratios D) Aβ42/Aβ40, E) Aβ42/Aβ38 and F) Aβ38/Aβ40 were plotted against the respective averages.
