# Supplementary material for: A two-step immunoassay for the simultaneous assessment of Aβ38, Aβ40 and Aβ42 in human blood plasma supports the Aβ42/Aβ40 ratio as a promising biomarker candidate of Alzheimer’s disease
Source: Alzheimers Res Ther. 2018 Dec 8;10:121. doi: 10.1186/s13195-018-0448-x (PMC6286509; doi:10.1186/s13195-018-0448-x)
Supplement: Supplementary file 2 — Aβ40 standard curves of the two MSD assay plates applied to analysis of the clinical sample. (PDF 283 kb) [file 13195_2018_448_MOESM2_ESM.pdf]

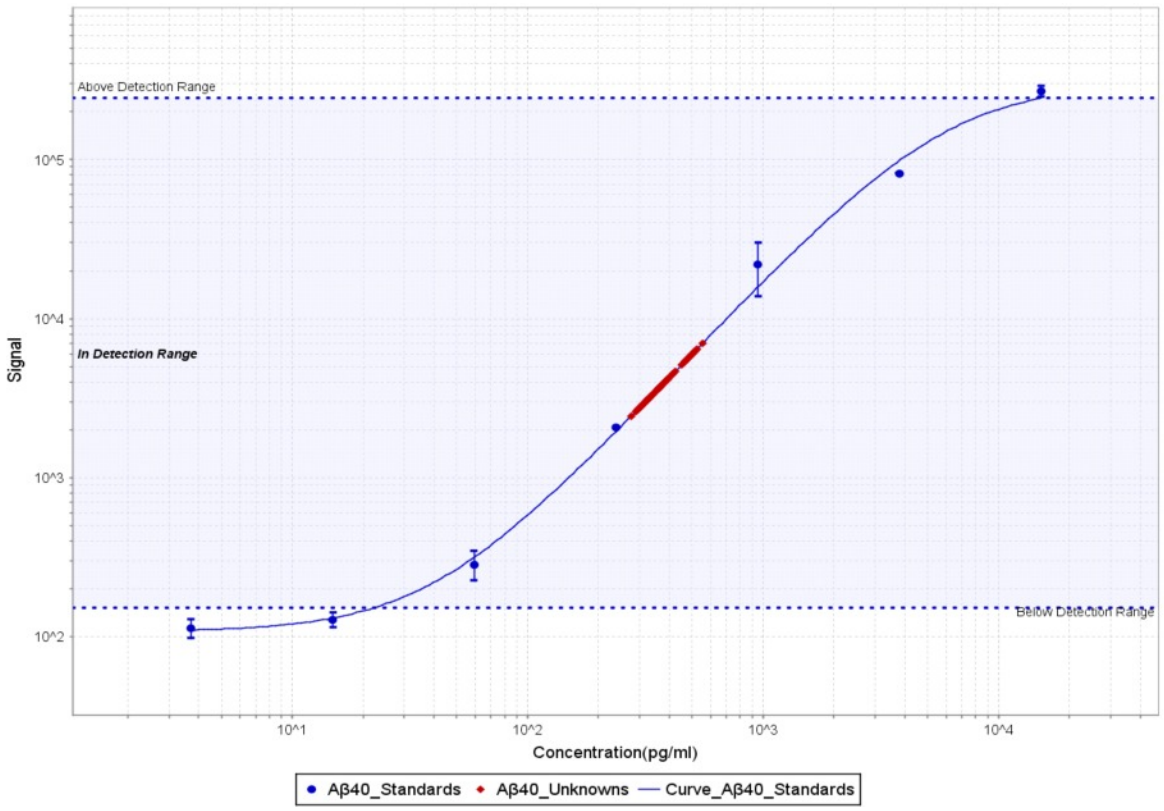

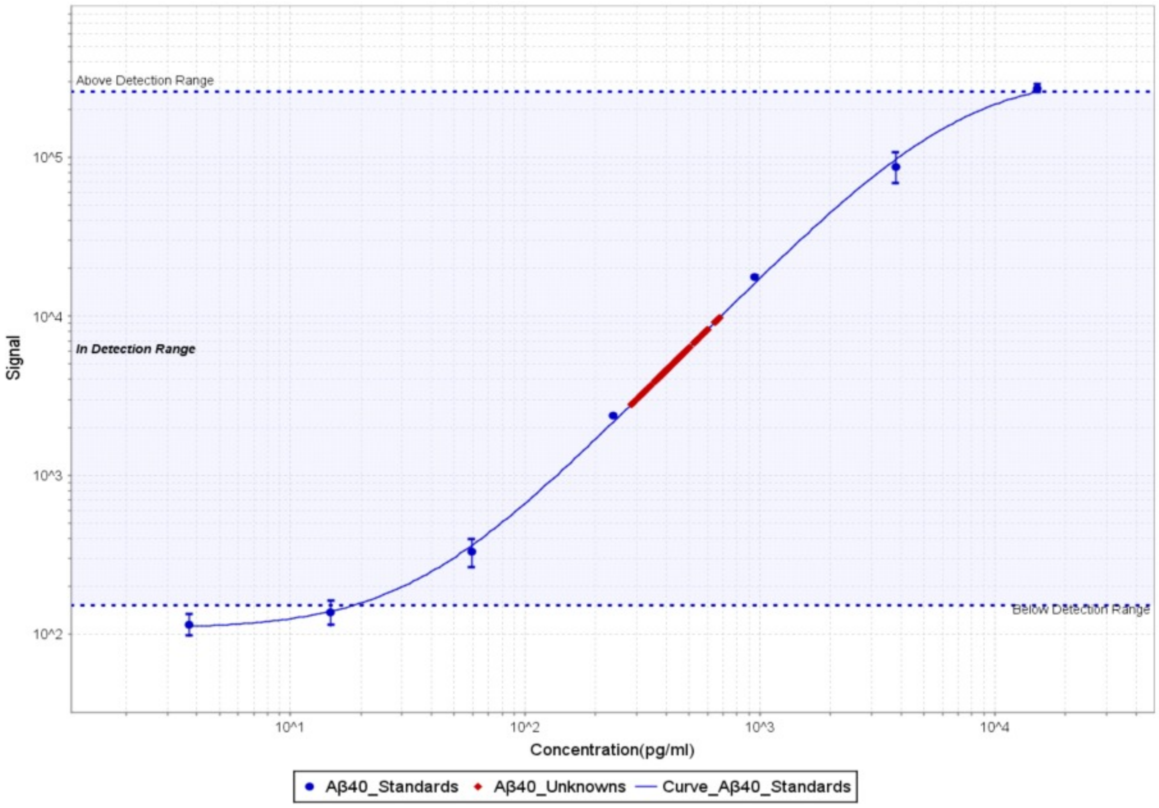

Additional Figure 2: Aβ40 standard curves of the two MSD-assay plates applied to the analysis of the clinical sample. The graphs show the signals of the four-fold dilution series of the Aβ40 calibrator peptide and the standard curves in blue. The signals obtained with the diluted IP eluates are shown in red. The bottom and top of the detection range are defined as the LLOD of the assay plate and the highest calibrator concentration, respectively.
